# Rewilding: history, intervention and the quest for immanence

**DOI:** 10.1007/s40656-026-00727-4

**Published:** 2026-03-19

**Authors:** Nuria Valverde Pérez, Òscar Castro García

**Affiliations:** 1 Institute of Philosophy, Spanish National Research Council (CSIC), Albasanz 26-28, 28037 Madrid, Spain; 2 Department of Philosophy, Autonomous University of Barcelona (UAB), Edifici B. Carrer de la Fortuna, 08193 Barcelona, Spain

**Keywords:** Rewilding, Functional approaches, Benchmarking, Historicity, Immanence, Ethical rewilding, Performative science

## Abstract

In recent years, rewilding theories and initiatives have gained momentum as a credible solution to the loss of ecological diversity and stability. However, rewilding remains a controversial theory that draws our attention to the multiple links between intervention, history, and the value of nonhuman capacity for self-organization. Tracing the history of practices and theoretical frameworks of some emblematic projects and proposals in this field, we focus on the shortcomings and theoretical challenges of functional approaches, including notions of functional equivalence, and the difficulties posed by counterfactual reference points. At the heart of this analysis are the contradictions that some of these approaches pose with the crucial goal of rewilding, which is, in principle, to preserve immanence and spontaneous organization. By way of conclusion, the recommendation to deepen reflection on the past for a genuine and ethically sound incorporation of the feral into everyday life is presented.

## Introduction

*Rewilding* is a concept considered to describe a biodiversity conservation strategy based on the cascade effects of “animal ecosystem engineers”—as well as an associated way of living (Monbiot, 2014). It remains a controversial concept since the definition of its restorative aims vary from preserving or increasing wildness in nature (among widespread discussion about what “wild” is); recovering ecosystem function; or producing self-sustaining ecosystems (Schulte to Bühne et al., 2022), which are in some respect at odds with each other, mainly regarding levels of commitment towards environmental restoration (Klop‐Toker et al., 2020; cfr. Pettorelli et al., 2018). Accordingly, practices related to it are also considered to be variegated and contradictory (Jørgensen, 2015).

However, despite this lack of homogeneity, it is supposed that all forms of rewilding share an ethos. Lorimer and colleagues (2015) considered that it was based on maintaining “or even increase biodiversity and reduce or reverse past and present human impacts by restoring more functional ecosystems.” To the extent that rewilding supports the creation of novel ecosystems, a broader consideration of this *ethos* foundation would be based in reorganizing biota and ecosystem processes “to set an identified social–ecological system on a preferred trajectory, leading to the self-sustaining provision of ecosystem services with minimal ongoing management” (Pettorelli et al., 2018). Despite obvious content differences, both definitions entail several epistemic problems: how do we thematize the notion of function, the role of history in its understanding, and the consequent ethical implications.

Rewilding as an ecological and political strategy is a reflection about causing harm, about regretting this harm and trying to reverse it. Intrinsically, it conveys the discussion about the possibility of damage reversibility, the role and place of the memories of harm and the memories of futures past^1^ that often puts us beyond the nature/culture divide. It also foregrounds questions about *what i*s being harmed, and what is *being harmed*—as well as other parallel issues: whether damaged entities or complexes are harmful (and in what cases); how do we consider “repair” or restoration works; and how can we deal with what is “beyond repair”.^2^

In principle, being able to point out what is damaged as “damaged”, means that we have some idea about its “normal” performance, its defining and essential features. [A position that depends on an idealistic and teleological epistemic framework.] Also, that there is evidence or (eco)memory of a time where such unharmed existence was viable, in the sense of being able to resist the constrictions and limits imposed by other beings and non-organic elements of the system where it developed. If rewilding implies a commitment to reduce human ecosystem management to a minimum (Carver, 2019; Perino et al., 2019; Schweiger & Svenning, 2020), it is because damage is essentially linked to dissolving or hindering symbiotic and life sustaining interactions that can be *easily* sustained and managed by the entities involved in them. In other words, excess of human control or management impacts the range and possibility of non-humans to persist by defining their own needs, giving place to emergent relationships, or to become otherwise. Reversing damage through the process of rewilding is related to making possible again such *easiness* without sacrificing the entities’ potential to resist, self-organize and self-preserve in its relationship with other beings.^3^

In the process of being, entities are always becoming, reacting, coping, organizing, and self-restructuring—though there is a limit to such dynamics and to plasticity, so extinction or permanently damaged existences are a constant possibility (Malabou, 2012). Because organisms and systems do cope, intermingling with one another, we must be cautious in defining our interventions towards damaged individuals, declining species, metastable systems, and emergent microdynamics so as not to frustrate the alternative futures they harbor, whose possibilities we not always foresee. Or, in Canguilhem’s (2008) terms, to be sure that we do not sacrifice genesis to function (139), nor individuals to types (123ss). Creating the conditions for that to be so, has led restoration movements towards discussing the old problem of immanence or conservation of process.^4^ From this perspective, proposals such as Lorimer’s (2015) to establish an affective logic in our relationship with the environment expose the limits of conservationism conceived solely as “the instrumental desire to secure the delivery of ecosystem services” (9). Lorimer’s performative conservationist approach relies on immanence understood as “an ecological assemblage composed of a single substance and characterized by emergent properties, rather than transcendent essences” (28). This puts an end to human exceptionalism, but also provides the basis for our understanding of cohabitation with other beings in terms of mutually sustained spaces for self-development.^5^ In this sense, caring for the ability, right and power of self-determination and self-preservation of any creature can be considered to be at the core of rewilding ethos, to the point it sustains non-human agency restitution and fights crude anthropocentrism (De Jonge, 2004). Justification for biodiversity also hinges on articulating this caring.

However, immanence implies perseverance in being, but the manifestations of this perseverance are tentative and even superfluous (Canguilhem, 2008), that is, they are not oriented towards accomplishing a function, and thus perseverance cannot be totally defined in functional terms. [Either because perhaps we ignore other possible functions, because functions may be emergent, or because we are epistemically biased]. However, the idea of function and functional restoration are crucial in rewilding for several reasons.

To some extent functional and teleological approaches are embedded in the structure of scientific policies. Despite the insistence of rewilding approaches on rescuing natural agency and thus open-ended interventions, a “desired outcome” is often the goal of the rewilding project (Corlett, 2019; Du Toit, 2019). To be assumed as a collective desire, this goal must be presented as *achievable* through a selected intervention, *beneficial* to humans in political, social and/or cultural terms, and truly aimed at *restoring* wild nature. Still, the bifurcation between ethical ends and epistemic means—comprising the logical, observational, experiential and material tools human beings can deploy to reach such ends—raises the question of the extent to which human intervention to protect such ecosystemic and biological perseverance is compatible with the notion of function. To what extent is it useful—in epistemic and justice terms—to define harm reversal based on a functional approach?

Herein lies one of the difficulties of rewilding: defining the intervention, asserting the ethical content associated with respect for non-human agency without diluting it into inaction or negligent suspension, or threatening what is aimed to be preserved. As Hans Jonas (1984) pointed out, thinking of ethical commitment beyond the punctual commitments of the strictly contemporary, immediate present implies thinking of our techno(epistemo)logies not as mere tools for restoring functions, but as enablers of viable futures and new imminently constitutive experiences of that which we cherish and serve as tracers of ethical principles as yet unknown.

Of course, many of the philosophical implications of this imperative are beyond the scope of this paper, which focuses on exploring how attempts to make reversion of damage a mechanical, functionally defined reconstruction tend to dissolve the historical content that affects both the production of harm and the ways of reverting it.

In the following sections we explore the tensions of rewilding theories and practices, to clarify how their epistemic cartographies deploy forms of intervention in agonistic connection with notions of immanence and restoration. In the first section, *Epistemics and functions*, we outline some of the difficulties that the notion of function represents when serving as a regulator of biological systems. In the two following sections, *Trophic function: Epistemic choices and relational ontologies* and *Pleistocene’s promises: From non-man-land to coexistence by ecological engineering* we will explore the epistemics of the notion of function in what is called “trophic rewilding”, to clarify its role in the ontology and ethics of rewilding. *Land abandonment and practices of care*, deals with some approaches to minimal intervention, offering a hint of the challenges and practices associated with non-intervention. The last section offers a series of conclusions.

## Epistemics and functions

Concerns for the restitution of the possibilities of emergence, variation and conservation of natural entities have historically been associated with the criticism of providentialist positions. That is to say, cohabitation with Nature as a creative agent (dynamic system) implied the end of any certainty about the indefinite “natural” preservation of each one of its productions or of a selection of them (particularly of human beings).^6^ The idea of preserving a space for such a creative force to develop without the constraints imposed by human pressure has always reflected our fear for its extinction and our distrust of the possibility of controlling it without defusing it.

To restore a nature that does not have as its *telos* the preservation of the elements that make up a manifestation of the ecological system in a given time places human beings in an ethical conflict. Like each of the natural productions, human beings work for their own preservation; but they must do so without considering that the rest of Nature’s manifestations have to bend to their own end. On the other hand, that natural resistance which does not easily yield to imposed *telos* is what enables human being itself to emerge; considered as a whole, Nature does not allow the establishment of hierarchical privileges among its productions. This apparent distancing from particular ends and this contingent genesis have served as a basis for the justification of the alteration of the environment aimed to the expansion of the species (as in bio(eco)engineering)—destruction being considered a “natural” trait for subsistence (De Jonge, 2004, 20ff). And when faced with that same destruction, affected people realise how dependent they are on the elements that have been destroyed. This polarization reflects the ambiguous position we assume about our responsibility in bearing the consequences of widespread ecosystem mismanagement and exploitation (Morton, 2016).

At the heart of our efforts to reconcile preservation and generative conditions is the notion of function, which is crucial for rewilding to model the role of extinct and extant species for managing their possible reintroduction in landscapes targeted for recovering. In some respects, this regulative approach tends to essentialize some links, and as it becomes the core of intervention design, it becomes a political and normative battlefield of understanding rewilding’s futures.

Lorimer and Driessen (2016) consider that functionalist approaches provide a more holistic view, though this does not mean that their resulting political ecologies are necessarily desirable, nor epistemologically sound.^7^ Notwithstanding, since the discovery of the hidden functions of an ecosystem is nowadays a scientific and technological enterprise, it is assumed that the confluence of technoscientific projects tends to produce better and more stable results “to secure desired systemic properties” (16). Functionality becomes the key word to glue up the notion of wilderness with scientific, technological and economic development, maintaining a managerial understanding of “nature’s services.” This last concept strains any notion of nature, as nature's services, creativity and diversity can differ greatly.

“Function” is a difficult and highly debated concept in biology (Wouters, 2003, 2005).^8^ Logically it is captured as the result of an analysis of the system and the explanation of the rationale that justifies the presence of each part, ontologically it is considered as a norm-establisher, and therefore—if we consider the process of living and individuation as dynamic—norm-changing. The problem is “function” is normally caught in *machinic* terms, that is, from the perspective of a desired (and biased) output that is considered to be there “as by design,” which reduces life in an operative ontology of being. It justifies the replacement of entities by other entities and processes—anthropogenic or not, based on biological, mechanical or cultural dynamics—that do not necessarily contribute more diversity to the ecological system (Du Toit, 2019) and that can lead to a reductionist vision based on the principle of interchangeability. That is, some entity can be substituted by another one as long as it accomplishes a similar function that has been epistemically privileged, considered to be “functionally equivalent”. This reification is highly problematic in ecological and biological systems, as there are parts of the system that accomplish no evident function, and are nonetheless inherent to it, since versatility, improvisation and tolerance to monstrosities are inherent to life (Canguilhem, 2008, p. 90). Moreover, when considering the prevalence of certain traits in an ecobiological system, if the function of one entity can be substituted by a similar function of another entity, the functional explanation dissolves, or weakens, as does the justification for the substitution (DiFrisco, 2017).

It is clear, though, that any system configuration has a plasticity limit beyond which it undergoes an ontological transformation: it becomes another type of system that works under a new configuration. “Services of nature”, thus, *also* designate qualities of a certain configuration of relationships that allow a series of system elements to exist. They are not just an output but a condition of possibility. Providing drinking water or clear air are “services” that affect more than one species, they are common goods. The links and relations that support the *configuration* of a system simultaneously allow movement, displacement and change without challenging or nullifying the configuration (Canguilhem, 2008). The task of “restoring or preserving functions” pivots on a liminal position between acting in support of a configuration and, by way of a deep transformation due to a recovering intervention, ousting it. Leaning one way or another depends on how we understand the biopolitics accompanying it. Or, how we understand anthropogenesis in a framework that seeks to give support to non-humanly controlled immanent processes.^9^

Canguilhem highlighted that the main effect of our willingness to understand systems as mechanisms—as “an assemblage of deformable parts, with the periodic restoration of the relationships between them” (2008, p. 77), hence as reversible processes—is that we replace a “political anthropomorphism” with a “technological anthropomorphism” (2008, p. 86). Nature becomes functionally shaped in the way human beings understand technological artifacts. Polyfunctionality, functional emergence, and sheer ignorance do not appear clearly, if at all, in the techno-anthropological landscape thus provided. The traces of contingency and unexpected desire disappear in favor of historically petrified needs and desires.

Rewilding confronts these tensions as differences over the notion of functionality arise when intervention is confronted with non-intervention. In the following sections, we will explore how the functionality works and its implication in the valuation of the intervention in the two main rewilding strategies: restoration of trophic functions and abandonment of the land.

## Trophic function: epistemic choices and relational ontologies

An essential part of the rewilding proposals focuses on trophic functions to configure the technological/mechanistic design of the intervention. The basic idea is that humans have displaced large mammals and predators, restricting the movements of large herbivores to limited spaces, preventing the distribution of seeds in wide spaces and distances.

On the contrary, the “landscapes of fear” (Laundré et al., 2010) produced by “*trophocracy*”—understood here as the power to modify the behavior of other species to the extent that they are considered prey—apparently guarantee this function. Trees felled by the action of megaherbivores bring decay and insects, attracting birds, and contributing to stabilizing soil temperature. In principle, introducing predators or large herbivores will trigger causal events that would sustain biodiversity. Thus, through specific intervention driven by anthropic interests, human beings hope to unleash this cascade of effects, relying on the functional stability of a system that, in human’s own opinion, is metastable and is subjected to such pressure that it is already crumbling, or degrading toward less plastic configurations.

According to this picture, trophic rewilding has been described as “an ecological restoration strategy that uses species introductions to restore top-down trophic interactions and associated trophic cascades to promote self-regulating biodiverse ecosystems” (Svenning et al., 2015).

The trophic function is a relational category, and as such it is historical. Nutrition is the effect of a process in context and time, so it is subject to change. Something becomes food contingently. Trophic chains are connected both with the ability of modifying prey’s behavior, and reinforcing the coexistence of herbivores, carnivores and omnivores, food vegetation, fungi, slime molds, microbiota, detritus, etc., or “the multiple trophic guilds” (Du Toit, 2019). A trophic chain oriented towards diversity fulfills the sense of “preservation” by allowing any being to intervene in its environment to produce food, giving rise to any entity’s immanent *conatus*,^10^ coexistence with and readaptation to the chaos that develops in its ecosystems.

Thus, the question centers on how the diversity of “competing” *conatus* in a given context can preserve ecological niches that evolve on non-linear environmental changes*.* For example, the reintroduction of predatory species can cause dramatic effects for communities of megaherbivores or mesocarnivores, given the need for new adaptive behaviors concerning a complete information domain of the locations of such predators (Delibes-Mateos et al., 2019; Tambling et al., 2015). In line with the dynamic nature of the trophic relationship, the prey–predator link can be reversed, for example, in the case of the reintroduction of lobsters to control the whelk population that had increased as a consequence of the extinction of lobsters in the area. (Delibes-Mateos et al., 2019, p. 356; see, for other examples of these inversions, Kaitala et al., 2021; Sánchez-Garduño et al., 2014; Wizend & Gasith, 2011).

To guarantee control over the “trophic chain,” the model used to reach the benchmark must consider the behavior of those “associated species” that are part of the world where human beings develop. Lower visibility taxa cannot be ignored (see Contos et al., 2021; Du Toit, 2023; Schindler, 2006). Neither can the social, epistemic and technological practices that support damaging behaviors and technological practices. Status quo may remain intact despite pernicious effects if we do not revise our ontoepistemic choices.

Jared Diamond (1983, 1986) has long ago drawn attention to the complexities of natural and field experiments. In many cases, trophic rewilding intervention incorporates a counterfactual dimension in the models, the comparison with a scenario where ecosystems evolve without human presence, placing them beyond the experimental continuum proposed by Diamond. Thus, Svenning et al. (2015) establish the measure of loss of diversity and, therefore, of the urgency in the intervention, based on the comparison between the “current state” and a speculative “natural state” or, as they put it, “present-natural”, defined as “the state in which a phenomenon would be in today in the complete absence of human influence through time.” (900). The speculative approach rests on the regime of *ceteris paribus* conditions*: other things being the same*, the impact of a variable’s intervention can be assessed and controlled. In this case, the human presence is suppressed and it is assumed that this will produce no noticeable effects on regularities between the associations or relationships that the entities of the system establish in accordance with the observation systems.^11^

These difficulties are not unique to ecology, but affect any dynamic and complex system of which it can be said, following Cartwright in her reflection on economics, that it “is a consequence of a mix of factors with different capacities operating in a particular environment. The mix is continually changing; so too is the background environment” (Cartwright, 1995, p. 279). The acceleration of climate change in recent years confirms the impossibility of keeping background conditions “the same” amidst the relational transformations (emergent and historically sedimented) undergone by any selected set of observable agents.

Precisely because what must be kept “the same” in order to make a comparison requires a collective effort and takes place in an environmental scenario in crisis, the statement of trophic rewilding is ample: should socioeconomic conditions (widespread technoscientific development, food production, housing provision to large populations) or services of nature (clean air and water, climatic stability for extant species) be kept “the same”? If it is the latter, then rewilding depends on an initial effort to preserve the minimum conditions under which feralization is possible (which can include sparing land as well as restrictions of energy use and technological overuse and colonization). If the socio-economic conditions must remain the same, then rewilding becomes an intervention enterprise to re-instantiate nature’s services through a system of functional (ontological) equivalences. [And, in this last case, rewilding adopts a strong technocratic approach that shapes its geopolitics].^12^ Thus, in this version it assumes the need of producing custom-designed elements in order to create the “machine” (Pemberton & Cartwright, 2014) for reestablishing equilibrium (or regularities) without interfering with technological development.

However, as in economic systems, arrangements and sustainment of the conditions of possibility cannot be planned on the basis of individual behavior of the parts, because defining the function of individuals does not produce regularities (Cartwright, 1995). This makes the outcome of any attempt at replacement or substitution uncertain. Consequently, the regularities and interactions easily produced and sustained by the individuals and communities involved become an object of concern and care.

The speculative exclusion of the human being from the targeted environment might have other consequences. By failing to recognize the scope, importance, and mutual influence between humans and other species, the rhetoric of these counterfactual “natural” benchmarks obscures past futures and options canceled by our biopolitical (infra)structures. The reflection on historical initiatives that favored a successful coexistence with the wild and that were aborted by other more advantageous biopolitical initiatives for geopolitical entities in a position of force is suspended.^13^

As the trophic function is complex and uncertain, species reintroduction can be considered an adventurous venture, even if there is evidence that reintroduction is a successful strategy to generate “net functional and phylogenetic gains” (Hatfield et al., 2022). Adventurousness is not necessarily a negative trait. It is the effect of the inverse relationship between ontological diversity and the possibility of creating robust models, between the richness of description and the limitations of functional definition and anticipation. Rewilding processes are said to be slow; time is needed to see the effects on environmental dynamics and the transformation of the landscape. In the case of Yellowstone Park, wolves were reintroduced in 1995. Three decades later, biologists are pleased with the effects of wolves on roaming and foraging elks, the willows and the beavers. But the “cascade effect that plays out between animals and plants […] will take decades of research to understand.” (Farquhar, June 20, 2021; see also Bakker & Svenning, 2018) Ecosystem services are always identified *post facto*, resulting from a narrative proposed through the initial model (Wise, 2011).

If initial results are encouraging, the evolution still remains uncertain. Global impact remains unclear, especially because experiments contemplate regeneration in limited spaces, unsuitable for serious consideration of any cultural and economic transformation and adjustment of human behavior. Additionally, rewilding processes are oriented to a fine-grained temporality that we cannot monitor; some immediate transformations only manifest themselves when they manage to scale, making them almost impossible to control. Thus, we are faced with a mismatch between our epistemic strategies, reinforcing models based on medium-term solutions and reliance on “nature’s services”, and the difficulty of assuming the different levels of temporality and the complex dynamics of diversity systems.

The inevitable idealization that trophic restoration models entail has yet to address another question: the “technoanthropology” question. This question arises more clearly when we face the complexities of de-extinction aimed at reestablishing landscapes that have their referent in the Pleistocene.

## Pleistocene’s promises: from non-man-land to coexistence by ecological engineering

Le labyrinthe du continu n’est pas une ligne qui se dissoudrait en point indépendants, como le sable fluide en grains, mais comme une étoffe ou une feuille de papier qui se divise en plis à l’infini ou se décompose en mouvements courbes, chacun déterminé par l’entourage consistant ou conspirant. (Deleuze, 1988, p. 9)

As a branch of trophic rewilding, a quite accepted proposal for planning in a more specific way is the Pleistocene model. A landscape previous to *Homo sapiens’* expansion, collective collaboration, and social organization is chosen to unravel the functional ecological dependencies that guarantee a high level of biodiversity in an allegedly stable ecosystem. By 2004, discussion surrounding the role human beings played in Late Pleistocene megafauna extinctions seemed to tip dramatically towards human responsibility. Human hunting associated with climate change was claimed to be one of the main causes of extinction process, and to have driven the dramatic extinction of about two thirds of North American megafauna (Barnosky et al., 2004; Sandom et al., 2014). The debate on the relative importance of *homo sapien* impact is still open, as is the question of the degree of biodiversity and functional loss compared with the situation 8000 years ago (Hartfield et al., 2022) Nevertheless, the Pleistocene remains a tempting reference point as a restoration benchmark, as it installs us in an “idealized wilderness” that is “free from human influence.” (Martin & Szuter, 1999) It is considered that the counterfactual approach provided by the Pleistocene benchmark offers a more distributed responsibility on the sustained extinction and, hence, the burden of restoration (Monsarrat & Svenning, 2022).

The landscape that human presence de-stabilized being chosen as emblematic for creating a feasible horizon of rewilding has many implications. The most obvious is that it is presented as a “second opportunity” for dealing with biodiversity, associated with the idea of no-man-land spaces where nature thrives—particularly strong in North America, where extinction was much more robustly connected to human presence than in Europe (Barnosky et al., 2004; Broughton & Weitzel, 2018) despite the boost of bison and other herbivores in the same period (see Martin & Szulter, 1999).

The “second opportunity” of the Pleistocene benchmark comes with a series of reversion and de-extinction promises. Sergey A. Zimov’s *Pleistocene Park* rewilding project took shape in 1989—the park was started and fenced in 1996. It envisioned recovering the mammoth tundra-steppe landscape in Siberia, testing the hypothesis that human hunting and not climate change was the prime cause of megafauna extinction; a way of exploring “the role that Pleistocene animals played in maintaining their own ecosystem,” and mitigating global warming by avoiding the release of carbon sequestered in soils of the former mammoth ecosystem. (Zimov, 2005; Zimov et al., 2006) Posed in these terms, Zimov’s project was one of the most ambitious of the ongoing rewilding enterprises, becoming increasingly interesting from 2006 onwards. It offered a single “solution” to two of the major scientific and political concerns about our environmental futures: stopping biodiversity loss and slowing global warming. Observations on methane release due to permafrost thawing began to upset the scientific community in 2008 (Mascarelli, 2009). At the same time, the difficulties in reintroducing large herbivores into the subarctic zone of north-eastern Siberia became apparent.

Despite enormous efforts, the last seventeen years the Zimovs have been unable to escalate the dimension of its natural experiment. By 2018, the Pleistocene Park remained a 20 km^2^ fenced plot whose herbivore population was far below the critical point required to make a decisive impact on their environment: 1 European bison, 4 musk-oxen (*Ovibos moschatus*), 8 yaks (*Bos mutus*), 9 horses (*Equus ferus caballus*), about 20 sheep (*Ovis aries*), and at least two reindeer (*Rangifer tarandus*) (Popov, 2020). Attempts to assess the effectiveness of Pleistocene rewilding projects for recovering the mammoth steppe landscape are discouraging (Reinecke et al., 2021). Since 2011, Sergey and Nikita Zimov’s scientific work stressed the connection between the Mammoth Steppe Ecosystem and the control of the cryosphere change (Zimov & Zimov, 2011; Zimov et al., 2012). During this period Zimov came into contact with George Church at the Workshop on *De-extinction projects, techniques and ethics*, organized at the National Geographic headquarters in Washington DC. Bioengineering—regardless of Sergey’s skepticism (*The Planet*, 2013)—appeared the ideal tool for achieving the objectives of the Zimov’s project, and Pleistocene Park the perfect excuse for raising funds to carry out a synthetic biology adventure focused on the recovery of the woolly mammoth.

Since populating the steppe with big herbivores to prove the hypothesis required a long time, the team at Pleistocene Park produced results that further backed their hypothesis. They used a Soviet tank with a mechanical arm in place of a proboscis, to simulate the behavior of mammoths in their ability to fell trees. With this first step into bio-objectification,^14^ the Zimovs diverted focus towards shortcuts taken not just to prove their hypothesis, but to attain actual results. They resorted to a schematic functional model that translated complex neurobiological synergies into a mechanical scenario that could be easily assumed. They established a point of contact with fundamental epistemic practices of bioengineering stemming from synthetic biology, thus making plausible a slippage from functional explanation to functional design. Church’s assertion that “biological organisms are programmable manufacturing systems,” (Church & Regis, 2012, p. 18) epitomizes the coalescence of functional attribution with the inherent susceptibility to intervention. Rewilding as a possibility of restoring agency in the wild collapsed in favor of a new ontological order. The question is, if synthetic biology can provide the means by which the functional targets of the Pleistocene model can be attained in a shorter time, should we not acquiesce to the new technology?

By 2015 some bioengineers—e.g. Vincent Lynch (University of Chicago) and Georges Church (Harvard University)—began exploring the possibilities of de-extinguishing mammoths, or at least, transfer their basic functional properties to elephants to provide a faster solution to the Pleistocene landscape. De-extinction is defined as “the generation of proxies of extinct species that are *functionally equivalent* to the original extinct species but are not ʻfaithful replicasʼ.” (Novak, 2018, emphasis added) These can be attained through different processes. The three methods recognized by the International Union for the Conservation of Nature (IUCN) are selective breeding, genome editing and cloning.

Between these three technologies are deep ontological differences. Selective breeding implies several consecutive generations that phenomenologically share the same flow of time (and, unless catastrophe occurs, a shareable environmental experience), but each individual shares only a part of the somatic history of its predecessors. Cloning also involves a succession, but the individuals of each generation share almost the same somatic history of its predecessor. Gene editing means a break in succession, even if the individuals involved in the production of the new entity share part of their somatic history. When gene editing involves already extinct animals and phenomenological time is discontinued, the meaning of a shared “history” crumbles.

The time leap that takes place both at accelerating a process of (neo)ontological production and re-establishing past dynamics—or, as Church and Regis (2012) describe, “replay[ing] scenes from our evolutionary past”—does not take the shape of a fold in Deleuzian terms. The epistemic Cartesianism of synthetic biology manifests itself in “the BioBrick approach and in the strategy of standardization, decoupling and abstraction” (Gramelsberger, 2020)—in the conviction that distinction between parts implies their separability. In fact, it is the high level of abstraction of the de-extinct mammoth that shocks the reader in the first place, and that allows the “functional equivalence” to be plausible.

The notion of “functional equivalence” which de-extinction resorts to is akin to the “substantial equivalence” used by the agro-food bioengineering industry to justify the commodification of genetically modified organisms (Whatmore, 2002; also, Newell, 2003; Tomlinson, 2002). As the second is allowed to bypass the many social, cultural and biopolitical transformations entailed by GMOs; the first also overlooks the biopolitics under the contraction of time and space promoted by synthetic biology. The selection of “traits” to be embedded in a suitable “host” is “blind and opportunistic”—as Church and Regis claim natural selection is—as “from the point of view of multilevel selection theory, there is no such thing as *the* one and only kind of trait that selection always promotes.” (Sober, 2007, p. 155) This remains true for synthetic procedures. Led by technological bias, economic interests and cultural understanding of “biosecurity” and harmonized existence; it is impossible to rely on the selection of a trait for stabilizing a landscape and its environmental system. The new entity would inevitably become obsolete, for technological, socioeconomic or cultural reasons. Sustained intervention is then required, further claiming that “the course of synthetic genomics will be under our own conscious deliberation and control.” (Church & Regis, 2012). Of course, this technology of de-extinction is also an extinction strategy.^15^ This dramatic departure from rewilding to new synthetic environments raises questions on what is the benefit of this superseding; who is the “we” behind “our own control”; what kind of property rights are given to the companies that bioengineered these entities; and what are our ethical obligations with these “proxies.”

As Gramelsberger (2020, p. 755) notes, the term synthetic biology obscures extreme genetic engineering in high-security laboratories. Even if many bioengineered entities can be produced today by the general public, this does not imply that there exists lesser concern surrounding risks (see Trump et al., 2020). Synthetic environments aim to produce an anthropomorphic tailored environment, not a tailored relationship between entities that adapt their environment to their own needs. A safe, viable environment is understood as coexistence with pre-harmonized entities [and in this sense limited on its possibilities to act autonomously or otherwise.] Transformed into appliances, biotechnological entities are stripped of immanence and expressiveness. Life becomes not just a Laborartifakt, but an Arbeitartifakt: a perfect tool for frictionless perpetuation of functional extractivism.

The question becomes whether these interventions, following extractivist economic models, can be vindicated from other economic approaches. Apparently, design as a criterion for understanding the possible forms of a viable environment and measures to harmonize coexistence disappear in land abandonment. Intervention adopts a different dimension in this process.

## Land abandonment and practices of care.

Land-abandonment—the ceasing of agricultural or industrial activities—would apparently be the rewilding strategy that avoids bias against non-human entities and agencies considered non-directly linked to human interests, and humanly defined services of nature. It is claimed that, despite producing significant returns in cultural services and common goods, it is an unsustainable strategy as it implies financial losses (Lorimer et al., 2015). However, land abandonment is an increasing socio-historical trend.

Socio-cultural, economic and political factors of land abandonment vary between countries, regions or farms, depending on prevailing circumstances. Driving forces for land abandonment have been the decades-long global increase in rural depopulation (Carver, 2019), climate change and soil deterioration.

Scholars assess and qualify the impact of land abandonment in wildness recovery through measuring feral nature based on distance to roads, settlements, light pollution and potential of natural vegetation—or, distance from human influence. Abandoned land in Europe defined in these terms could contribute to wilderness and, allegedly, to the consequent improvement of water cycles. But, equally, abandonment could lead to a loss of biodiversity (Lorimer et al., 2015; MacDonald et al., 2000), as many species depend on agricultural and anthropogenic landscapes. Hence, one of the problems faced by rewilding by land abandonment is not simply determining the species assemblages we want to protect (Carver, 2014; Queiroz et al., 2014), but how to define control and care to match rewilding requirements. In other terms, not any kind of ferality is satisfying.

The cultural component of landscapes can be intensely destructive, but it also—depending on what we consider “natural” and worthy of care—incorporates the baggage of our knowledge surrounding care and conservation of the environment. For example, the case of the uncoated channels excavated for water harvesting developed during the Al-Andalus period, used until the 1950s (Malpica Cuello et al., 2021). [This is just one case where a technology paved the way for fruitful coexistence and an aesthetic bond.] Hence, the material and historical context of the landscape weighs heavily when deciding the type of passive intervention.

This context determines how we understand what it means to be indigenous when justifying passive rewilding. The native landscape depends on local and traditional values, and the loss of agricultural and livestock practices implies the disappearance of certain landscapes (Cvitanović, 2017). Revegetation of abandoned lands erases traces of the humanized landscape, again challenging the anthropological perspective inherent in the episteme of temporality and authenticity invested in the notion of “native”. But it also threatens to suffocate the co-constructed and collective nature of living beings. In this sense, given that cultural components are not avoidable—especially considering culture as a general strategy of living matter (Stewart & Cohen, 1997)—and since rewilding is driven by an urgency and ethical distress but requires time for deep and stable transformations, we can resort to minimal interventions in small plots instead. Fukuoka (1978) offers an agricultural model based in *sympoietic* synergies—a “making-with” and not just a “making-through” (Haraway, 2016)—that would largely keep the landscape unchanged in its wildness, while keeping human intervention minimal.

Fukuoka’s agricultural model is called the agriculture of “do not do,” and is one method in which “rewilding lite” (Carver, 2014) or “agricultural rewilding” (Corson et al., 2022) can be attained. It aims to flow with the natural evolution of the autochthonous ecosystem and wait for nature itself to “open up,” offering fruits, cereals and vegetables generated without tilling land or pruning trees. It implies deep changes in sow seasons. Rye, oats, wheat and barley in autumn are sown by broadcasting while rice is still standing. A few weeks later rice is harvested and its straw is spread over the sowing fields, harvesting around May 20. Its growth cycle is slower than in traditional or chemical methods. Contrary to the impoverishment—or even sterility—of the soil caused by other methods this cultivation process produces its enrichment. For this reason, it is claimed that in a healthy environment, the insects that cause pests only affect the weakest plants, without acting on the rest of the orchard.

Fukuoka’s model is also an ecophenomenological development. Its hybridity between the natural and the technological is developed in a “*techné*” as an art of listening to nature (Hoły-Łuczaj & Blok, 2023). As an example of cohabitation through active adjustment to onto-epistemic co-operation, it is transformative for all the involved elements. Its practice changes both the landscape and our expectations towards nature as a resource. Since it implies a change in sociocultural habits and behaviors, control is not reduced to function surveillance, nor care to improved adjustment to environmental stress and enhancement of the “vulnerable other.” Remediation in this case is affected over human activity, highlighting the fact that intervention over our surroundings is always self-intervention.

But land abandonment has other dimensions that affect our understanding of responsible intervention or responsible suspension of intervention. Since the 90’s scholars such as P. Galison, have called for attention on the process of auto-rewilding undergone in zones that have been devastated by the effect of nuclear radiation that became, as he put it, *wasteland-wildness*. The Chernobyl exclusion zone is a clear example. The intervention of previous human dwellers has been suspended for biosecurity reasons, but the flora and fauna that dwell in the area, deeply, ontologically transformed by mutations, challenge our notions of responsibility and preservative action. What kind of control and care can we deploy here? What kind of listening to nature? How can historical knowledge of the sources of damage and dynamics of destruction be integrated in ethical human (self)intervention in this case? Is the spontaneous rewilding taking place in these areas more legitimate than the rewilding initiatives designed to promote a humanly defined, functionally designed landscape, with its consequent weeding out of species? As rewilding aims to rescue non-human modes of agency that respond spontaneously and inherently to the possibilities of the environment, the answer would probably be “yes”. But to qualify the increase or diminishment of the adaptive plasticity of these new entities requires a historical approach to the kinds of futures that have been left behind by the nuclear disruption. [Following, then, the same intuition that drives the Pleistocene projects, but considering the levels of irreversibility as a call to limit the types of interventions that block or drastically narrow the range of possible futures.]

Enabling new futures for these altered environments—proc(u/a)ring to avoid the anthropogenic conditions that would lead to further degradation—while preserving the agency of their elements, involves confronting the historical and political choices that underlie this new ferality. A new ferality in which new environmental forces take the upper hand in shaping the features of entities, making it more difficult to think about immanent principles of self-organization, or, as Simondon put it, of plastic adaptations *of the environment* to the individuation process. Would such reversal of ecosystemic dynamics even allow us to think of rewilding?

## Conclusions

Rewilding as strategy is the expression of our fears, hopes and epistemic limitations. Exploring the many ways in which we are attempting to produce or trigger a more autonomous, regenerative environment out of damaged and menaced landscapes unavoidably forces us to rethink the conceptual, onto-epistemic cartographies that connect and give meaning to attempts of nature preservation.

It is clear that *rewilding* is not coextensive with preserving a “primeval” nature or “properly natural” entities. Nor can it be reduced to ecomodernist expectations of controlling ecosystemic productions by selecting functional traits, in a renewed version of a human-oriented providence. And, of course, human intervention must be in the picture. In a world permeated by politics, this is not only inevitable, but legal practice and economic power are crucial to sanction or curb damage to ecosystems. That intervention, however, must not be that of a taskmaster or shepherd (Morton, 2016), but that of an element aware of the damage and of the possibilities open to other paths.

It is also clear that the futures of such preservation—either in design or in its continuities—imply a forceful incorporation of the past as expression of alliances, manifestation of violence and indifference, or ways of successful cohabitation that remain embedded in current symbiotic and opportunistic ecological projections. Developing an “ethics of immanence,” (an ethics of the possibilities of what now becomes) cannot be resolved by consensus with the already “present presences,” but requires the presence of the past considered as the past of knowledge, affections, and trauma, the past of entities affected and those affected by the past. Certainly, some rewilding practices privilege specific past landscapes and ecosystems; but this loyalty to a specific past does not necessarily mean a sound political and ontological justification for this choice. Rewilding concerns our preoccupation with a loss, a broken link with the world, as Deleuze says. This link is not about restoring a future that has already passed— restoring an extinct landscape—but about believing in coexistence with more than human worlds that resist our notions of order, control and management. (Wynne-Jones et al., 2020) It implies ontologies that counter traditional and prevailing notions of human providence and stewardship.

No design strategy aimed at establishing a niche for ecological stabilization will be able to bring us back into a relationship with the wild. Nor will empirical observation of the survival strategies of living beings, however valuable, be able to put us on the road to this reestablishment. In fact, the return of feralness must emanate from a relearning to cohabit with it. This means that such reestablishment is impossible without a suspension of the privileges that humans arrogate to themselves in accessing and exploiting resources based on long-term depletion forecasts. Re-learning to cohabit with the feral will be, above all, an exercise of historical imagination, since what we are facing is precisely the absence of ferality. An exercise that requires an initiative that has as its starting point not the experimental logic, nor that of discovery, but that of procurement. A search that confronts something that was already there and speaks from afar. A search that takes care not of what we once had, but of what we never reached.

From an ethical perspective, the demand for rewilding does not have as its horizon the perpetuation of the human being as a biological entity dependent on a highly reliable system. Its horizon is the possibility of human experience understood fundamentally as an experience of the unresolved, of the uncertain. Politically, the uncertainty of continuity has fed aggressive policies of foresight, accumulation, and exclusionary survival. The quest for immanence at the core of the ethical requirement of preserving wildness is jeopardized precisely by this resistance to acknowledge our common future, not as species, but as intersystemic entities. Entities whose future cannot be planned, but can be sustained on a daily basis since intervention is conceived not as oriented towards a democratically assessed future, but as the labor of spontaneous mutual recognition of the non-fungibility of entities and the arbitrariness of dispensability.

## Footnotes

1 We borrow Koselleck’s (2004) expression. “Memories” here does not refer solely to *human* memories, but also to archaeological, material traces that informs our notions of extinction, change and sustainability.

2 Acknowledgment of what is “beyond repair”, in the sense of being unable to be restored to a previous state, feeds discussions about novel ecosystems and planned ecosystem services restoration.

3 Putting the notion of ease at the heart of reversing harms fills the request to give something back in terms of freedoms (see Monbiot, 2014), and gives some criteria to better discriminate the harms produced and the ways to reverse a state of affairs. However, such easiness, which respond to an ethical demand, is not effortless.

4 We use the term immanence in a Spinozist sense, filtered through the vision of Deleuze (1968) and the political approach of Bove (1996), which implies the relationality of elements (being each of them linked by relationships of cause and effect), the lack of ontological hierarchies between them, the need for variety, and the possibility of the destruction or extinction of any of these elements the moment they lose the capacity to resist the dominating, expansive action of any other. The term imply that no entity is a part detached from a totality, but a mode of an attribute or modification of a substance; as a consequence, none can be “imperfect”, hence “toutes les formes d’être s’affirment sans limitation” (Deleuze, 1968, p. 71), that is, they possess inherent force to exist and to act. Whereas resorting to the term “autonomy” could led to the supposition of disengagement from the environment, “immanence” and its correlation with desire and joy (hilaritas) in a Spinozist framework highlights interdependence and co-dependence (see Sharp, 2011). It implies the ethical dimension of acknowledging the structurally affective dimension of a “natural” relational system where distinctions between human and non-humans do not design antithetic but complementary ways of being.

5 Under an immanentist approach inspired in Spinoza, such space and “space making” should not be understood as the production of mutually exclusive spaces as much as the recognition of a (co-dependent) community within which there is a vibrant ontological diversity. In accordance with the idea that each element of this system aspires to develop its potential or capacity to exist according to its needs, the fullness of that potential is found in the simultaneous satisfaction of the potentials of the elements in such relation (Spinoza, Eth. IV, 18 demonstation; Bove, 1996, pp. 112–113; this is so even in the philanthropic posthumanism defended by Sharp, 2011. Cfr. De Lucia, 2019).

6 The eighteenth-century preoccupation with the variations of natural species will result this creative Nature (Ehrard, 1994) that cannot submit to the limits of preserving its own products: a conception whose moral implications would be developed by the Marquis of Sade (Klossowski, 2002 , pp. 168–170 [Esquisse, c.3]), who assumed part of Spinoza philosophy.

7 Thus, the auroch back-breeding project carried out by the Heck brothers in Nazi Germany naturalizes hunter-forester as a “beneficent keystone species” that guarantees the optimal functioning of the ecosystem (8), in part because they use science to legitimize their myths. Contemporary rewilding initiatives as the Tauros Program and Rewilding Europe—which, according to the authors, share a environmental biopolitics of decoupling with the Hecks’ project—also use myth, enchantment, aesthetics and intuition, but authors suggest that their use of myth as a mean to engage (and work) with diverse experts and publics (17) could favour a more proactive and progressive political ecology. This progressive character is linked to “a growing sense of the need to adapt to uncertain and unprecedented futures through nurturing the potential for wildness” (10), even if the article considers that Tauro Program and Rewilding Europe hardly acknowledged the bio- and geopolitical consequences of creating land areas decoupled of human presence and activity. On the other hand, it can also be said that the Uruz Project—a splinter movement of the Tauros Program—which they consider to be reductionist and mechanistic because it resorts to transcendent archetypes based in genetic evidence (7, 14), makes a similar use of science as a tool for justifying the hermeneutic circle (as noted by the authors) in which this use is embedded.

8 On the distinction between functions and functional explanations see (McLaughlin, 2004). However, most often the description of what an agent does is also its explanation. And, of course, the description depends on the technological means of observation and registering (Wise, 2011).

9 Herein lies the fracture between conservationism and ecomodernism, that notwithstanding is part of the rewilding movement (Karlsson, 2020; Lorimer & Driessen, 2016).

10 Spinoza defines conatus as “the effort by which each thing strives to preserve its being” and equates it to its essence. (Ethics, III, 7) It is the immanent organizing principle that sustains individuality and its disposition towards the environment.

11 Through this counterfactual strategy, a comparison is established that gives dimension to a potential loss of diversity. [Showing, in this case, that without these extinction factors—mainly Homo sapiens (899)—carnivores, mesoherbivores, and megaherbivores would probably enjoy much greater diversity today, and that the range of megaherbivores would be much more extensive. This does not mean that the loss of diversity has not occurred, but rather that the validity of this assessment is limited as it operates in an ideal scenario. The prescriptive force of this comparison, however, is evident in that it serves as a yardstick for measuring the impact of trophic rewilding. Svenning et al. point this out: “The potential ecological impact of trophic rewilding *should increase with the degree to which megafauna have been impoverished*. We provide a global estimate of such deficits (Fig. 1) by comparing the current species richness for three key terrestrial megafauna functional groups with their estimated species richness *given the present climate, but in the absence of Late Pleistocene and Holocene extinctions and extirpations*” (2015, p. 901, italics are ours). Note that according to the caption of Figure 1, it should say ‘estimated species richness *today*’.

12 Rewilding moves in a gradient between “dark anti-modernism” and ecomodernism. The contemporary ecomodernist approach shows more confidence in technological development for making nature serviceable (see Lorimer & Driessen, 2016), though the increasing diversity and nuanced discussions about urban rewilding and non-decoupling approach to wild(er)ness open new options.

13 A good example could be the alteration of the silo and watercourse system by the English empire in India by forcing the forward sale of Indian crops to the metropolis in the context of unfavorable climatic conditions (Davis, 2002).

14 We borrow the term from Holmberg et al., (2011), Bühler and Herbrand (2022).

15 See the discussions about the gene drive caused by the release of genetically engineered Aedes aegypti mosquitoes in Florida, aimed to suppress the population of this variety. https://www.nature.com/articles/d41586-021-01186-6.
